# Supplementary figures and images for: Aberrant cell segregation in the craniofacial primordium and the emergence of facial dysmorphology in craniofrontonasal syndrome
Source: PLoS Genet. 2020 Feb 24;16(2):e1008300. doi: 10.1371/journal.pgen.1008300 (PMC7058351; doi:10.1371/journal.pgen.1008300)

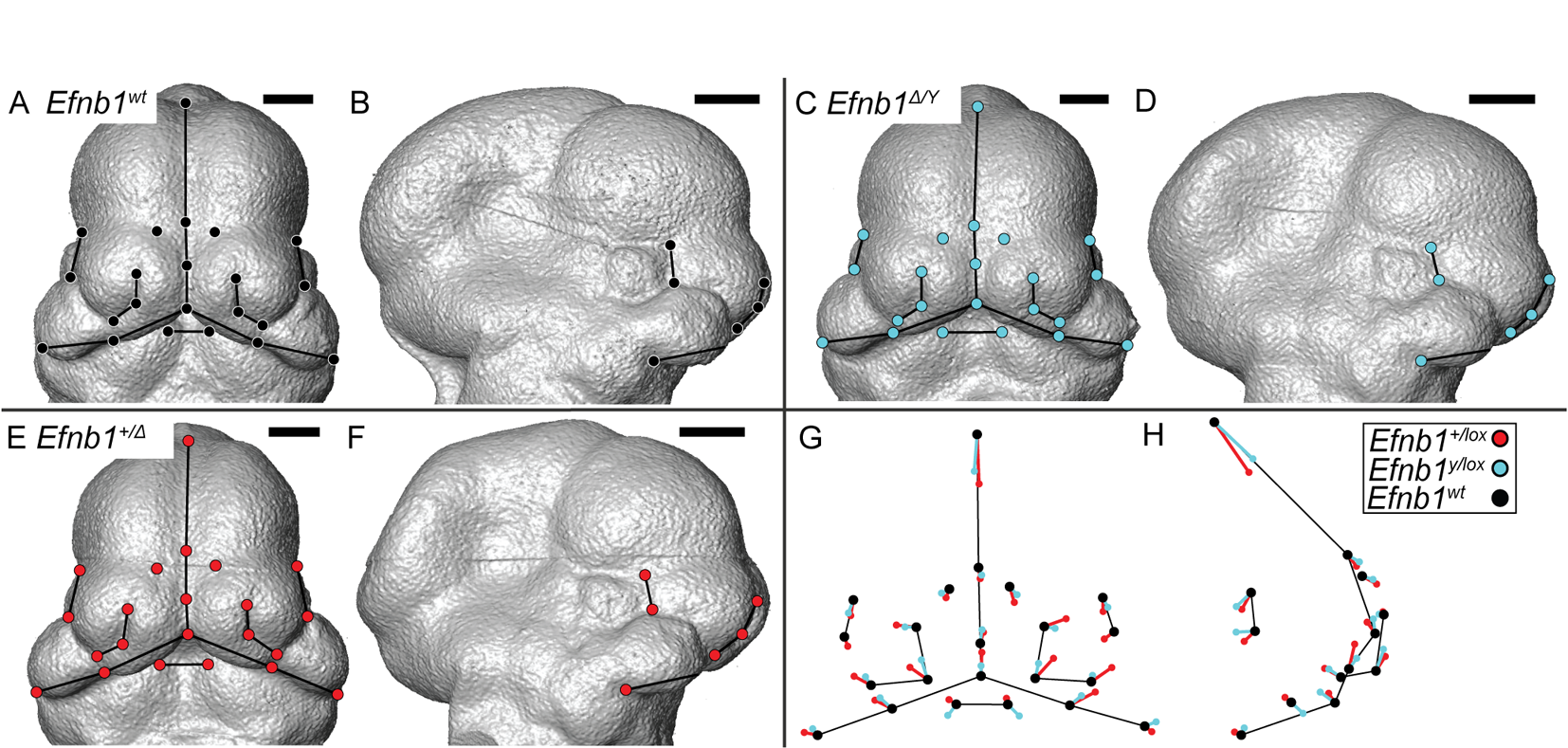

Supplement: S1 Fig — (A-F) Facial landmarks identified on representative Efnb1wt (A-B), Efnb1Δ/Y (C-D), and Efnb1+/Δ (E-F) E11.5 specimen surfaces. Scale bars, 500 μm (G-H) Common facial shape effects of Efnb1/Δ/Y (cyan) and Efnb1+/Δ (red) cyan genotypes on facial landmark position, compared to Efnb1wt (black) from the anterior (G) and lateral (H) views. The lengths of these shape difference vectors are magnified three times to allow for easy comparison. Thin black lines are placed for anatomical reference. (TIF) [file pgen.1008300.s001.tif]

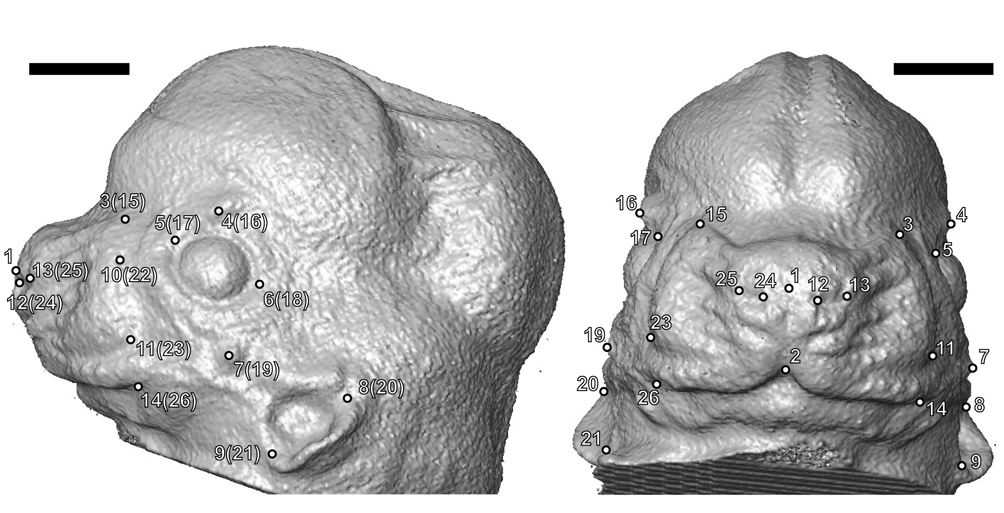

Supplement: S2 Fig — Facial landmarks used in morphometric analysis of E12.5-E14.5 samples, based on definitions found in S3 Table, identified on lateral (left) and anterior (right) views of a representative E13.5 wildtype specimen. Scale bars, 1000 μm. (TIF) [file pgen.1008300.s002.tif]

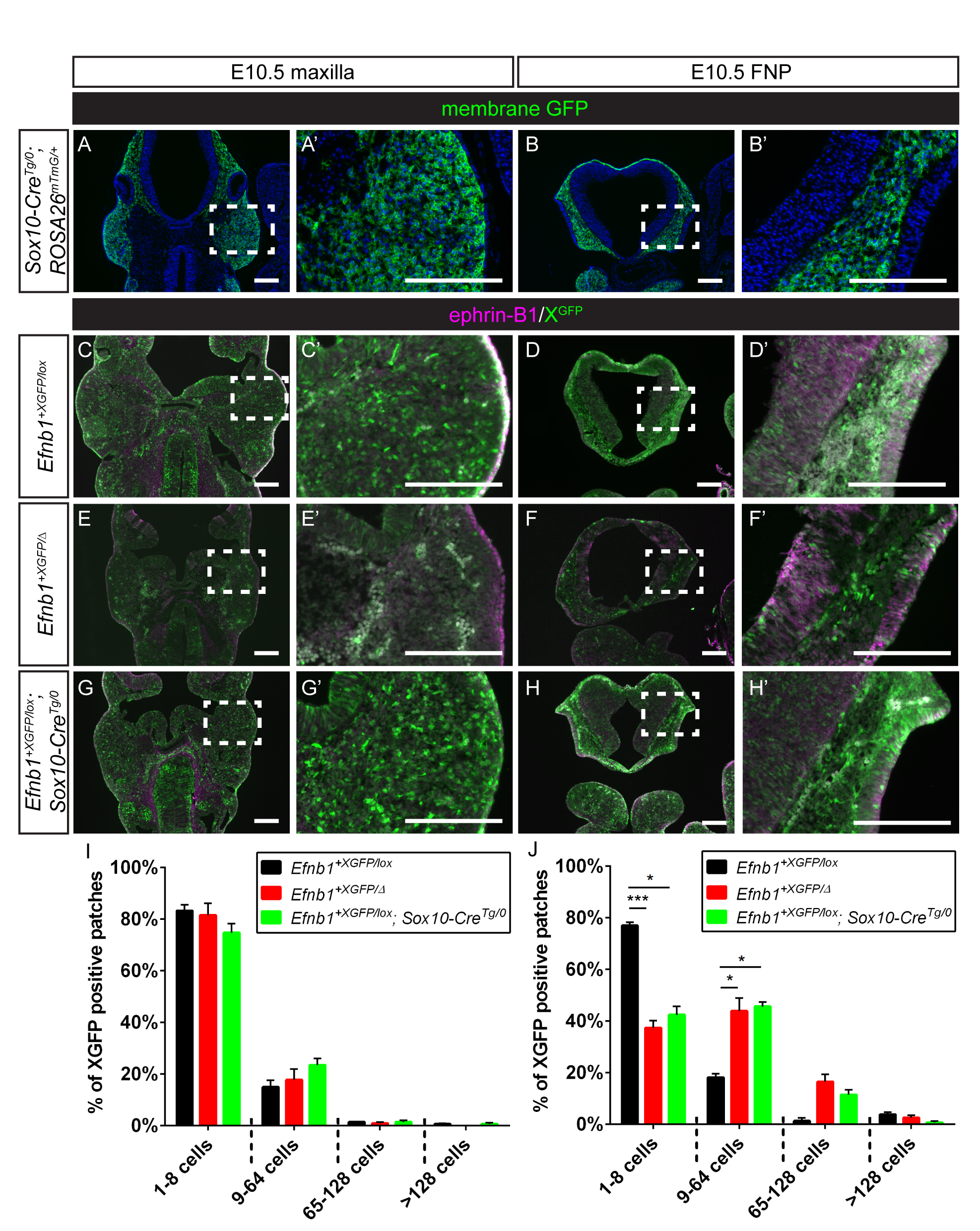

Supplement: S3 Fig — (A, A’) Sox10-Cre drives recombination in the NCC-derived MXP mesenchyme and (B, B’) frontonasal prominence (FNP) of Sox10-CreTg/0; ROSA26mTmG/+ embryos at E10.5. (C, C’) Efnb1+XGFP/lox control MXP and (D, D’) FNP demonstrate a fine-grained mosaic pattern of XGFP expression at E10.5. EPHRIN-B1 expression is not strong in the maxillae but has begun to be upregulated in the FNP at this stage. (E, E’) Likewise, neural crest-specific Efnb1+XGFP/lox; Sox10-CreTg/0 heterozygous embryos demonstrate a fine-grained mosaic pattern of XGFP expression in the maxillary prominences at E10.5, indicating that segregation is not carried through from migratory NCCs. (F, F’) The FNP of E10.5 Efnb1+XGFP/lox; Sox10-CreTg/0 heterozygous embryos shows a small amount of segregation, visible as patches of GFP expression and non-expression, likely because EPHRIN-B1 has begun to be expressed in the FNP at this stage. (G, G’) The maxillae of full Efnb1+/Δ (recombination mediated by Actin-Cre) are also not segregated at E10.5, but segregation can be seen in the neural tissues of these embryos. (H, H’) Segregation is visible in the developing LNP and in neural tissues of full EPHRIN-B1 heterozygotes. Scale bars, 200 μm. (I) Distribution of percentage of XGFP-positive patches of various sizes in the E10.5 maxilla. Column height represents means of the distributions across all sections measured for a given genotype, error bars represent S.E.M. (J) Distribution of percentage of XGFP-positive patches of various sizes in the E10.5 FNP. Column height represents means of the distributions across all sections measured for a given genotype, error bars represent S.E.M., *, P<0.05.; **, P<0.01; ***, P<.005; ****, P<.0001. Number of embryos analyzed is presented in S1 Table. (TIF) [file pgen.1008300.s003.tif]

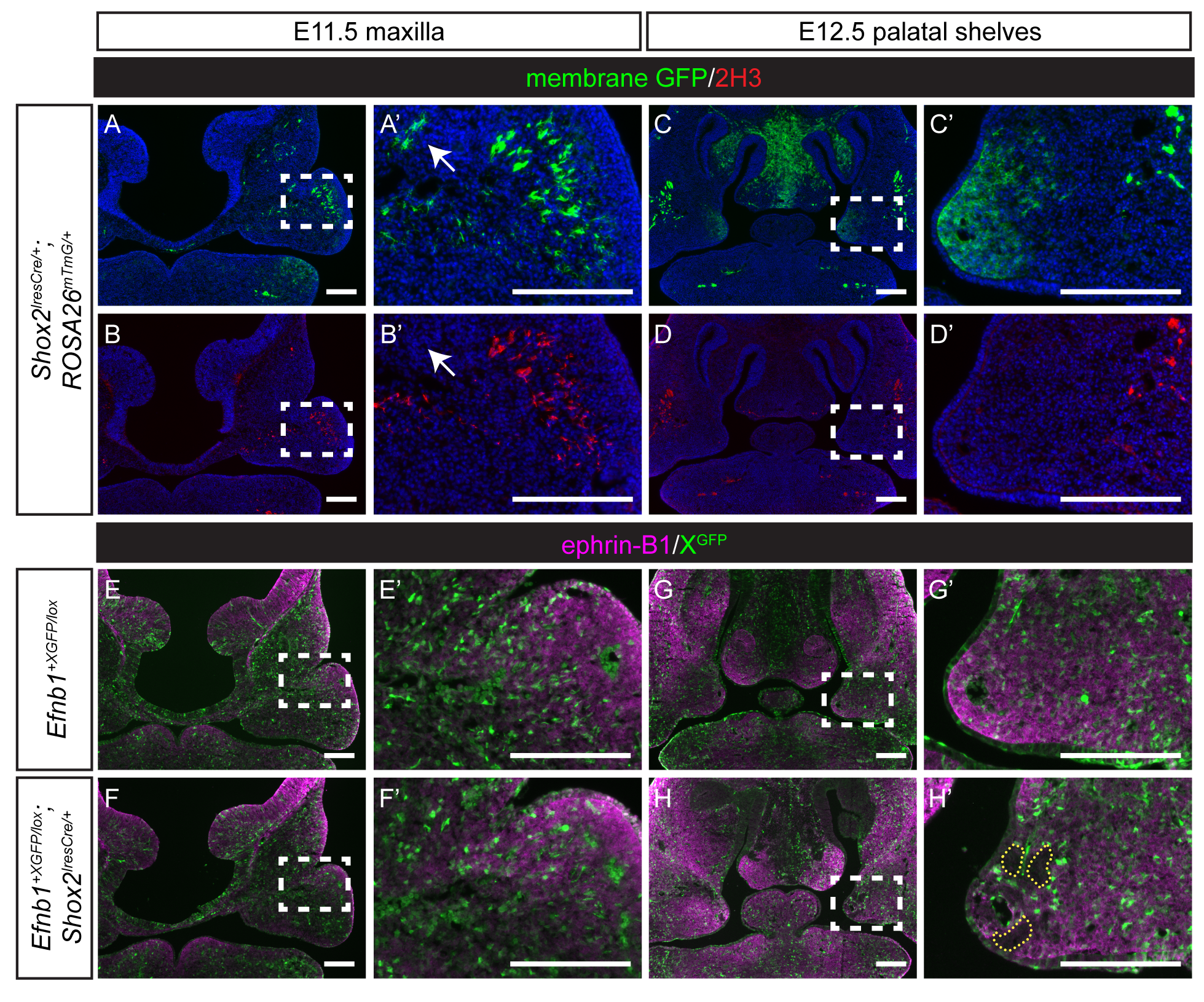

Supplement: S4 Fig — (A, A’) Shox2IresCre drives minimal recombination in the maxillary prominences of Shox2IresCre/+; ROSA26mTmG/+ embryos at E11.5. (B, B’) Most membrane GFP-expressing cells also express neurofilament (2H3) and are likely nerve cells of the maxillary trigeminal ganglion; only a few mesenchymal cells have undergone recombination at this stage (white arrows). (C, C’) By E12.5, Shox2IresCre/+; ROSA26mTmG/+ embryos express membrane GFP in the palatal shelf mesenchyme as well as (D, D’) in the nerve cells of the maxillary trigeminal ganglion. (E, E’) At E11.5, the maxillae of Efnb1+XGFP/lox control and (F, F’) Efnb1+XGFP/lox; Shox2IresCre/+ heterozygous embryos are indistinguishable; both genotypes demonstrate a fine-grained mosaic pattern of XGFP expression in the maxillary prominences, indicating that no cell segregation has taken place. (G, G’) At E12.5, control palatal shelves show a fine-grained mosaic pattern of XGFP expression. (H, H’) Small patches of EPHRIN-B1/XGFP expressing and non-expressing cells (dashed yellow lines) are visible in the palatal shelves of Efnb1+XGFP/lox; Shox2IresCre/+ heterozygous embryos at E12.5, demonstrating that post-migratory neural crest cells are also subject to segregation mediated by EPHRIN-B1 mosaicism. Scale bars, 200 μm. (TIF) [file pgen.1008300.s004.tif]

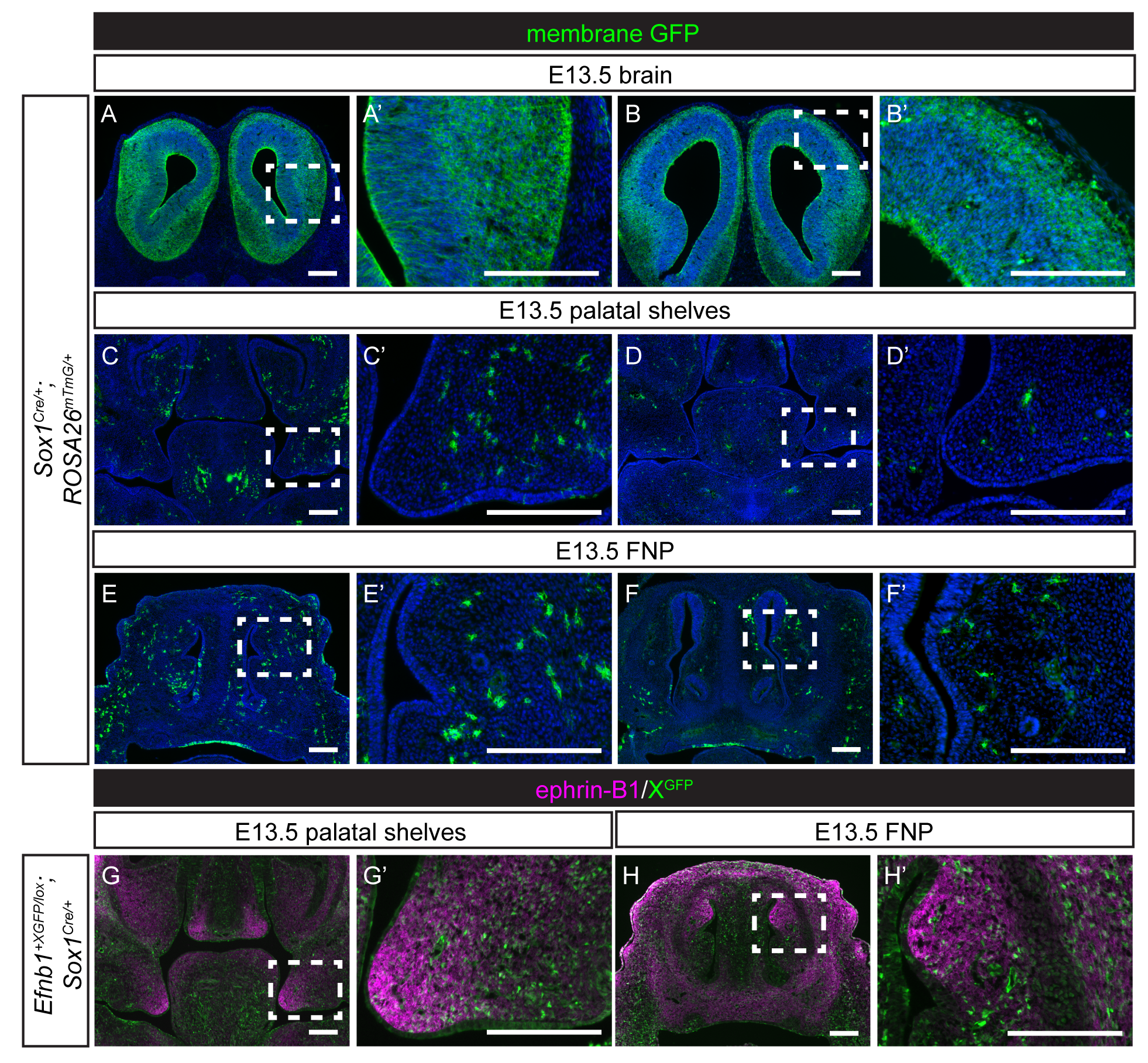

Supplement: S5 Fig — (A-B’) Recombination of the ROSA26 locus in two different Sox1Cre/+; ROSA26mTmG/+ embryos leads to widespread membrane GFP expression throughout the brain at E13.5, but minimal membrane GFP expression in (C-D’) anterior palatal shelves or (E-F’) anterior frontonasal prominence (FNP). (G,G’) Immunofluorescence against EPHRIN-B1(magenta) and XGFP (green) demonstrates that mosaicism in early neural progenitor cells mediated by Sox1Cre does not drive segregation in neural crest-derived craniofacial structures such as the anterior palatal shelves or (H, H’) FNP. EPHRIN-B1 expression (magenta) and craniofacial morphology appear normal in these embryos, indicating that neural progenitor cell segregation is an independent process. Scale bars, 200 μm. Number of embryos analyzed is presented in S1 Table. (TIF) [file pgen.1008300.s005.tif]

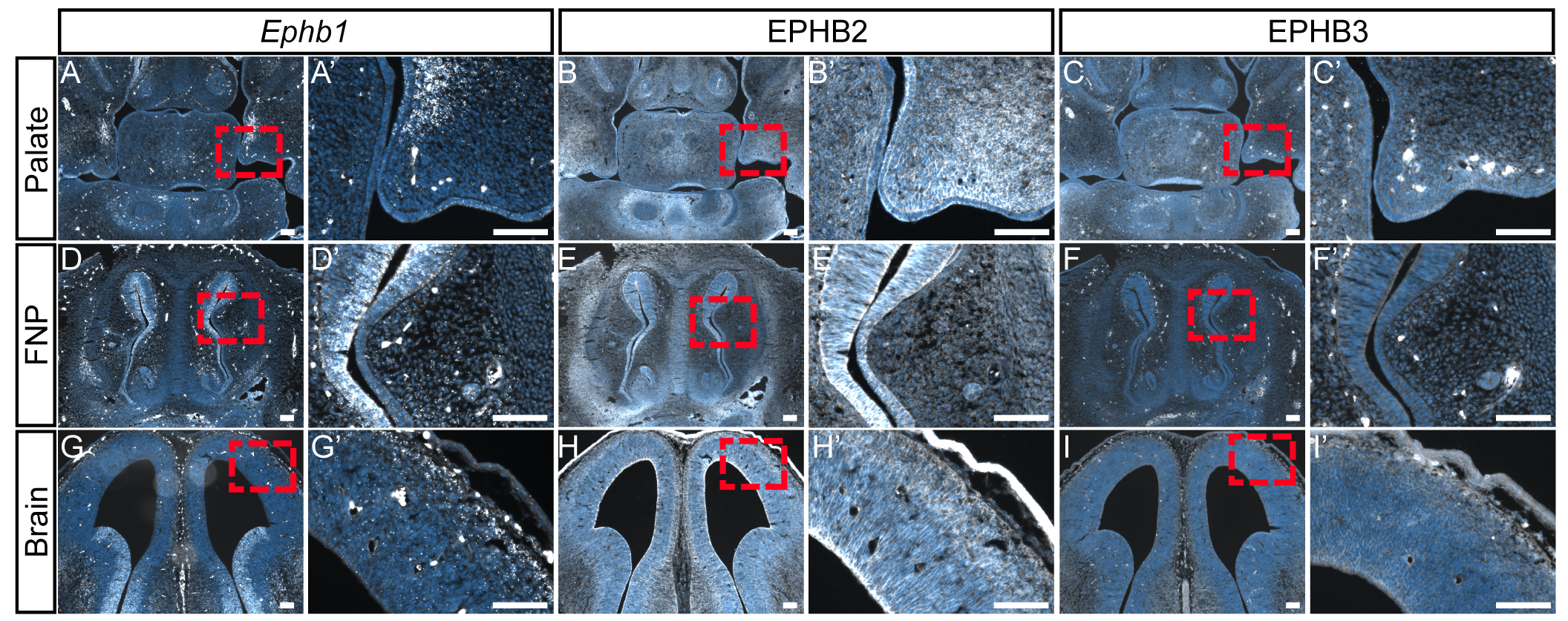

Supplement: S6 Fig — RNAScope in-situ hybridization analysis of Ephb1 expression in the (A, A’) secondary palate, (D, D’) FNP, and (G, G’) brain of E13.5 embryos. (B-C’) Immunofluorescence staining against EPHB2 and EPHB3 in the secondary palate, (E-F’) FNP and (H-I’) telencephalon of E13.5 embryos. Scale bar, 200 μm. (TIF) [file pgen.1008300.s006.tif]

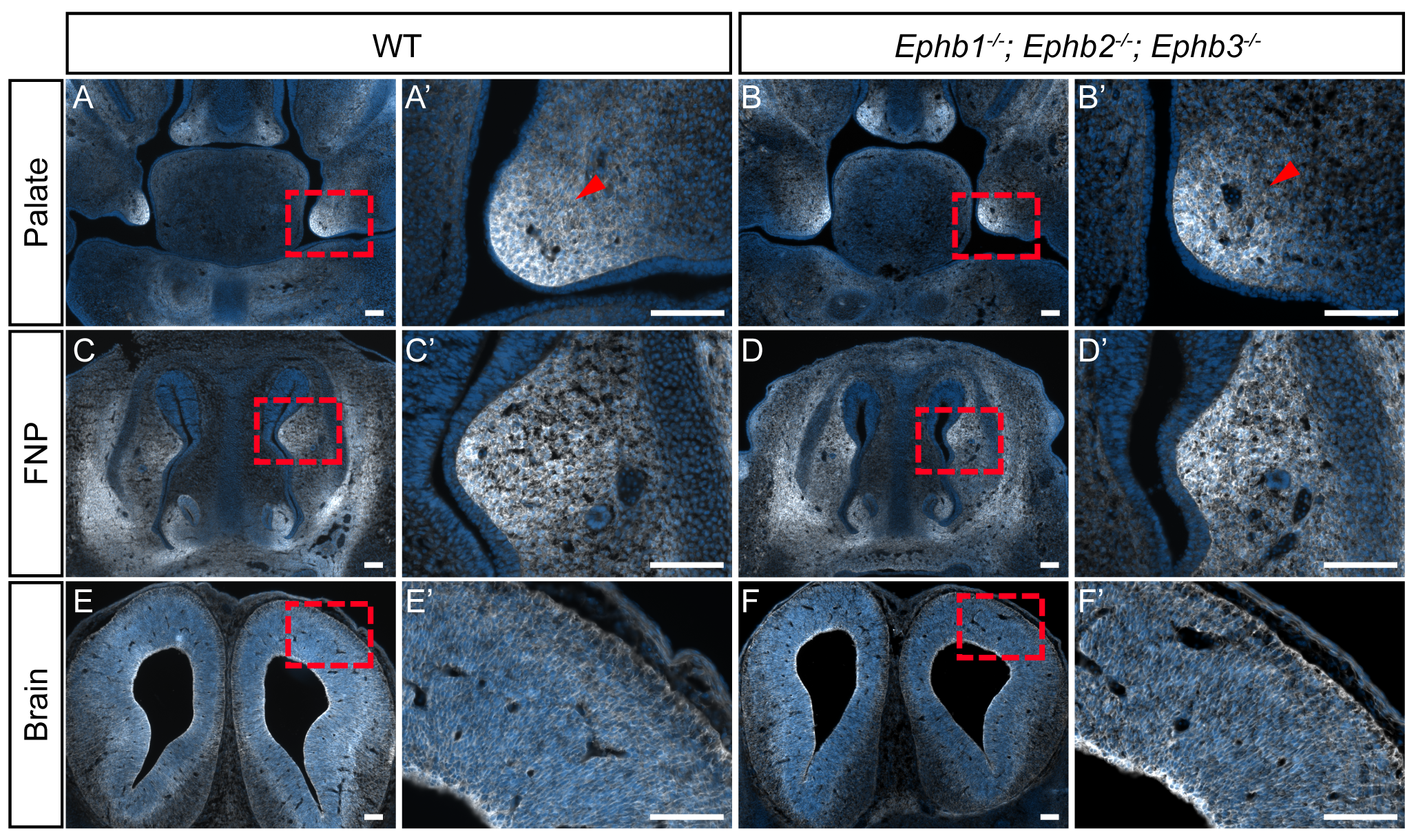

Supplement: S7 Fig — Immunofluorescence staining against EPHRIN-B1 in (A, A’, C, C’, E, E’) control and (B, B’, D, D’, F, F’) Ephb1-/-; Ephb2-/-; Ephb3-/- compound mutant embryos does not reveal overt differences in distribution, though the shortened shape of the secondary palatal shelves in Ephb1-/-; Ephb2-/-; Ephb3-/- leads to a reduction in the size of the area usually expressing EPHRIN-B1 in the secondary palate (red arrowheads in A’, B’) (A-B’). Scale bar, 200 μm. Number of embryos analyzed is presented in S1 Table. (TIF) [file pgen.1008300.s007.tif]

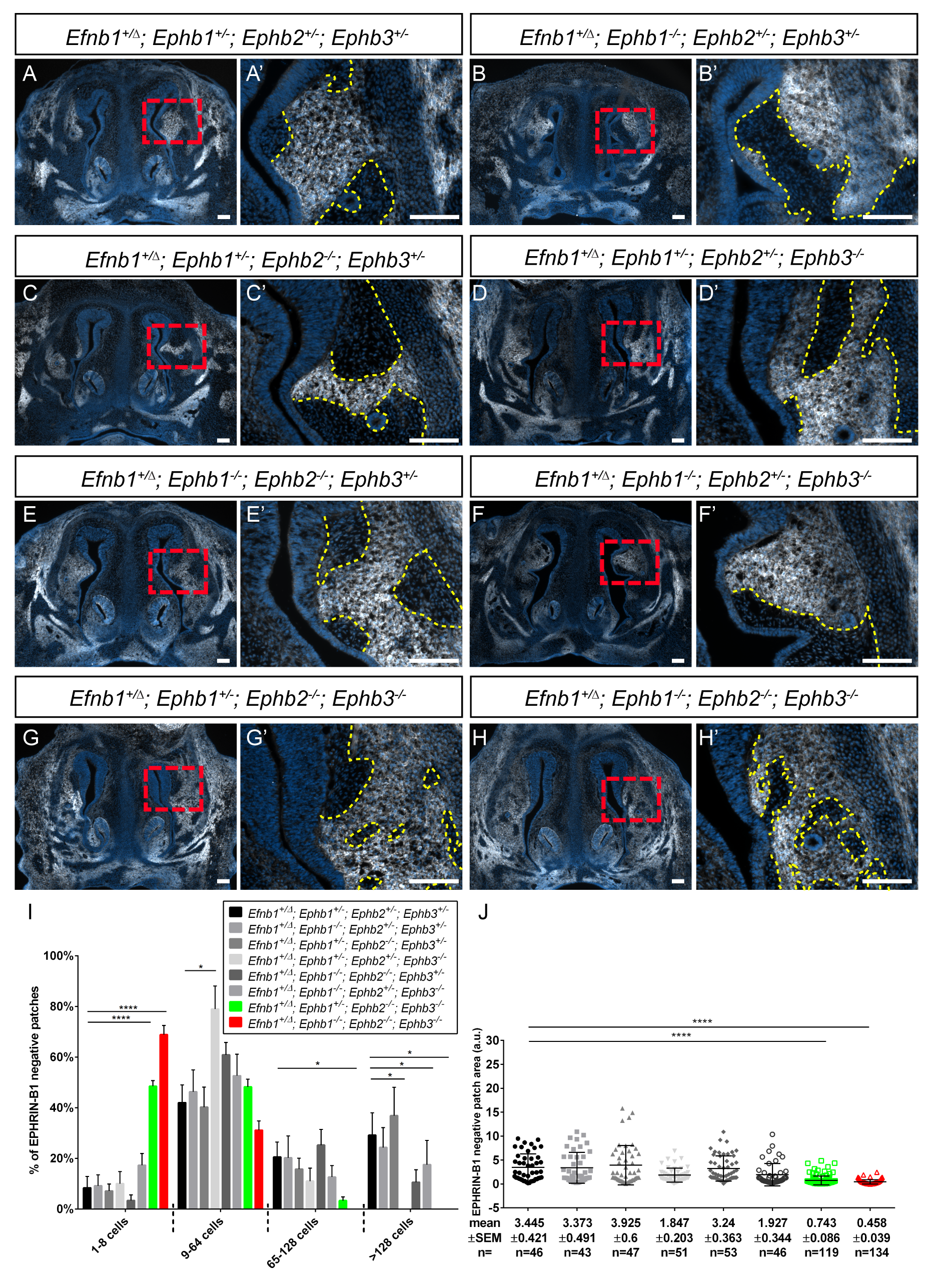

Supplement: S8 Fig — Frontonasal processes of E13.5 embryos harboring compound loss of Ephb1-3 receptor genes in combination with Efnb1+/Δ heterozygosity with specific genotype combinations shown. Immunostaining for EPHRIN-B1 expression (white) and DAPI (blue) is highlighted with a yellow dashed line at high magnification to demarcate cell segregated patches. (A-F) Compound loss of some EphB receptors does not reduce apparent EPHRIN-B1-driven cell segregation, with a relatively small number of large patches of cells observed. (G, G’) Compound loss of EphB2 and EphB3 receptor resulted in smaller patches, with greater intermingling of EPHRIN-B1 positive and negative cells. (H, H’) Loss of all known EPHRIN-B1 receptors (EphB1, EphB2, EphB3) also resulted in loss of cell segregation, but with the persistence of small patches of EPHRIN-B1 negative cells. Scale bars, 100 μm. (I) Distribution of percentage of EPHRIN-B1 negative patches of various sizes. Column height represents means of the distributions across all sections measured for a given genotype, error bars represent S.E.M., *, P<0.05; **P<0.01; ***P<.005; ****, P<.0001. (J) Patch sizes represented as scatterplots. Horizontal bars represent means, and error bars represent S.E.M. *, P<0.05; **, P<0.01; ***, P<.005; ****, P<.0001. Number of embryos analyzed is presented in S1 Table. (TIF) [file pgen.1008300.s008.tif]

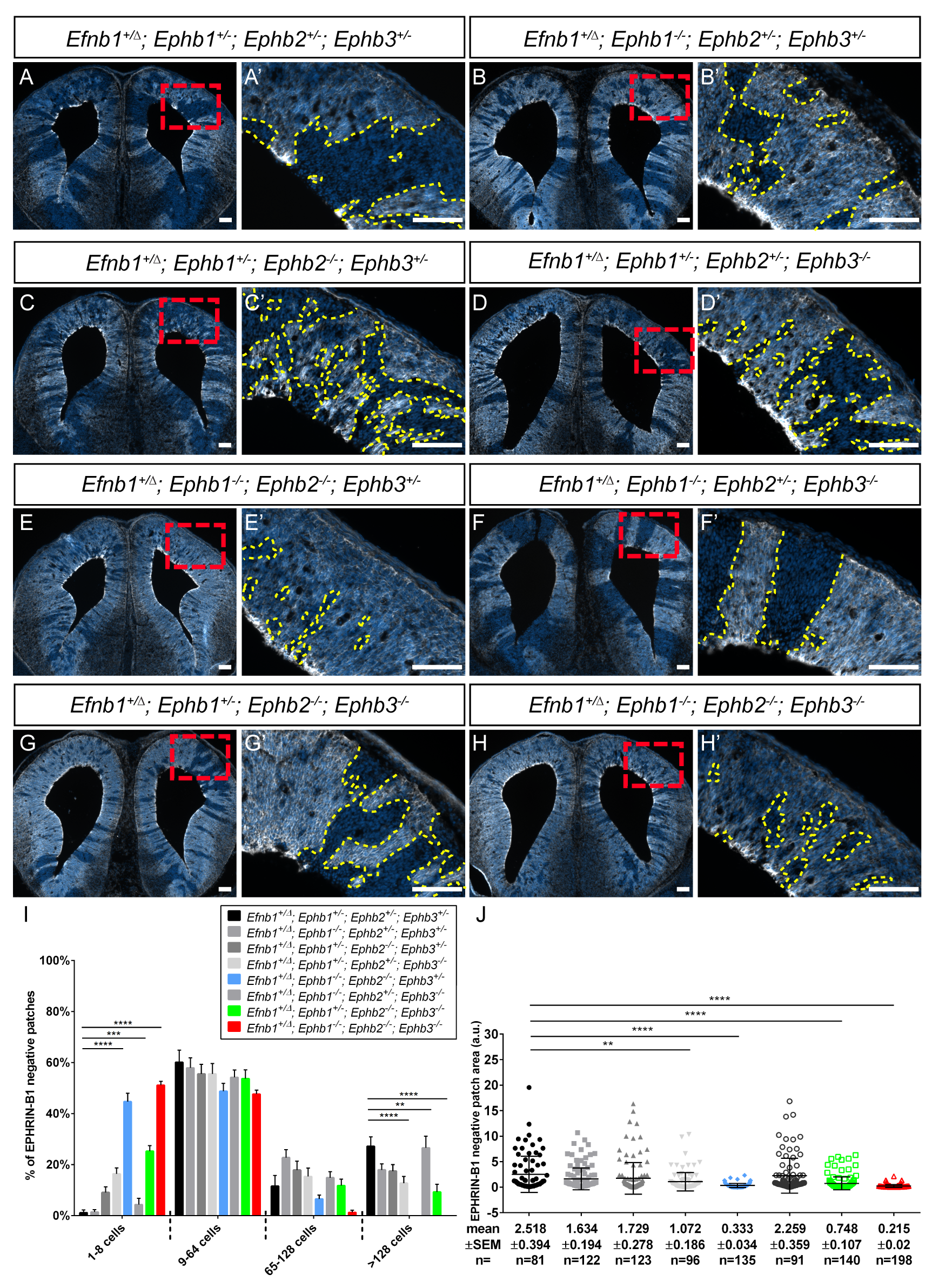

Supplement: S9 Fig — The telencephalon region of the telencephalon of E13.5 embryos harboring compound loss of Ephb1-3 receptor genes in combination with Efnb1+/Δ heterozygosity with specific genotype combinations shown. Immunostaining for EPHRIN-B1 expression (white) and DAPI (blue) is highlighted with a yellow dashed line at high magnification to demarcate cell segregated patches. (A-D) Cell segregation was robust, but variable in its pattern with haploinsufficiency for various EphB receptors. (E, E’) Compound loss of EphB1 and EphB2 consistently resulted in a dramatic reduction in cell segregation, whereas (F, F’) compound loss of EphB1 and EphB3 exhibited no apparent reduction in cell segregation and (G, G’) compound loss of EphB2 and EphB3 was intermediate. (H, H’) Complete loss of all three EphB receptors resulted in a dramatic reduction in cell segregation that was similar to compound loss of EphB1 and EphB2. Scale bars, 100 μm. (I) Distribution of percentage of EPHRIN-B1 negative patches of various sizes. Column height represents means of the distributions across all sections measured for a given genotype, error bars represent S.E.M., *, P<0.05; **, P<0.01; ***P<.005; ****, P<.0001. (J) Patch sizes represented as scatterplots. Horizontal bars represent means, and error bars represent S.E.M. **, P<0.01; ****, P<.0001. Number of embryos analyzed is presented in S1 Table. (TIF) [file pgen.1008300.s009.tif]

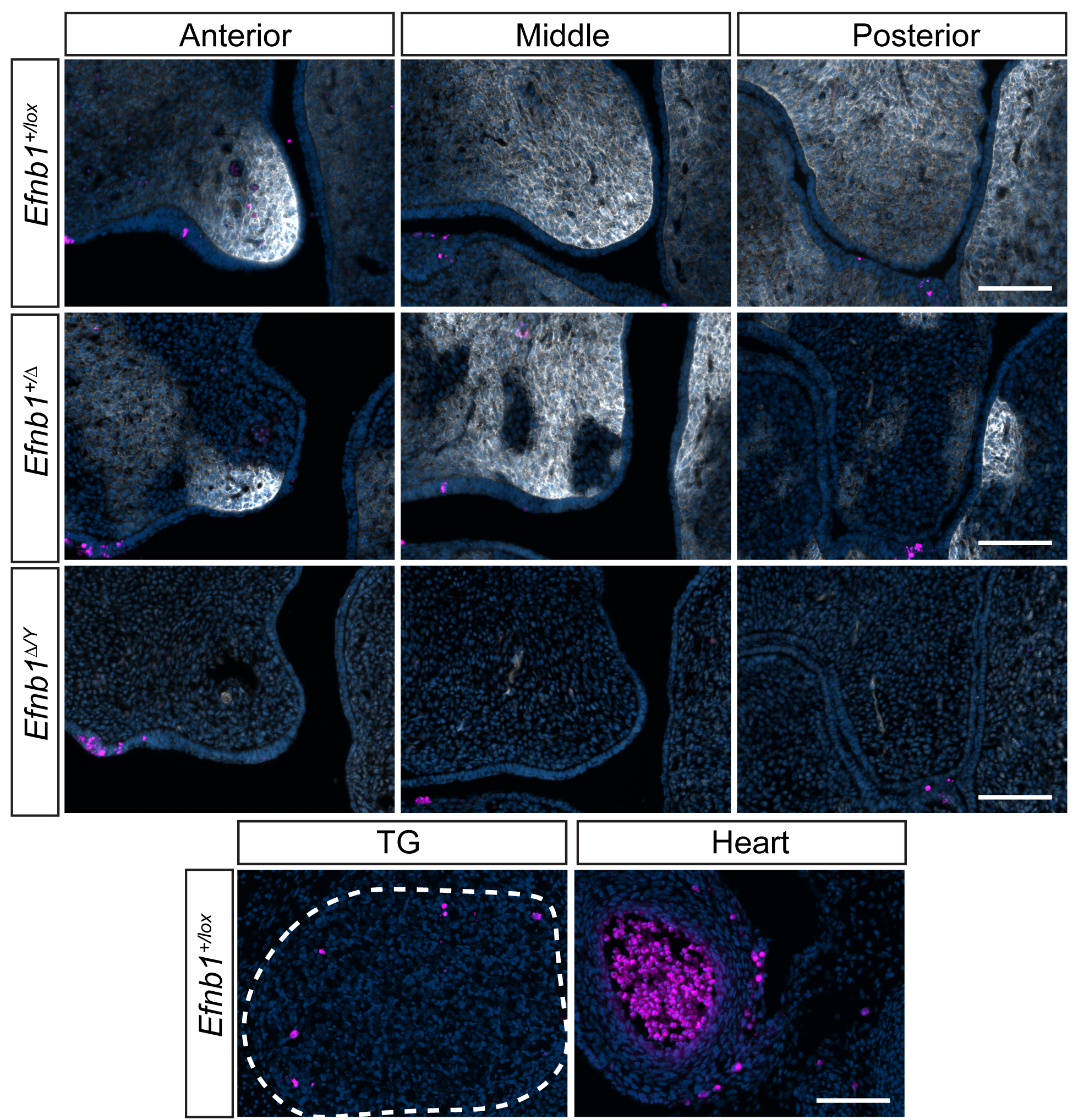

Supplement: S10 Fig — Immunofluorescence staining against EPHRIN-B1(white) and cleaved caspase 3 (magenta) reveals that little apoptosis is found in the secondary palate mesenchyme in control or Efnb1 mutant embryos. Scale bar, 200 μm. TG, trigeminal ganglia Number of embryos analyzed is presented in S1 Table. (TIF) [file pgen.1008300.s010.tif]
