# Supplementary material for: Aberrant cell segregation in the craniofacial primordium and the emergence of facial dysmorphology in craniofrontonasal syndrome
Source: PLoS Genet. 2020 Feb 24;16(2):e1008300. doi: 10.1371/journal.pgen.1008300 (PMC7058351; doi:10.1371/journal.pgen.1008300)
Supplement: S2 Table — (DOCX) [file pgen.1008300.s012.docx]

Table S2. Antibody information for immunofluorescence (IF)

| **Primary Antibodies** | **Source** | **Catalog #** | **Dilution** |
| --- | --- | --- | --- |
| EPHRIN-B1 | R&D Systems | AF473 | 0.2 μg/mL |
| EphB2 | R&D Systems | AF467 | 1:10 |
| EphB3 | R&D Systems | AF432 | 1:20 |
| GFP | Abcam | ab13970 | 1:500 |
| 2H3 (neurofilament) | DSHB | 2H3 | 2 μg/mL |
| **Secondary Antibodies** | **Source** | **Catalog #** | **Dilution** |
| Donkey anti-rabbit Alexa Fluor 488 | Jackson IR | 711-165-152 | 1:400 |
| Donkey anti-mouse Cy2 | Jackson IR | 715-225-150 | 1:400 |
| Donkey anti-chicken Cy2 | Jackson IR | 703-225-155 | 1:350 |
| Donkey anti-goat Cy3 | Jackson IR | 705-165-003 | 1:300 |
