# Supplementary material for: Aberrant cell segregation in the craniofacial primordium and the emergence of facial dysmorphology in craniofrontonasal syndrome
Source: PLoS Genet. 2020 Feb 24;16(2):e1008300. doi: 10.1371/journal.pgen.1008300 (PMC7058351; doi:10.1371/journal.pgen.1008300)
Supplement: S3 Table — (DOCX) [file pgen.1008300.s013.docx]

**Table S3. Landmarks for E12.5-E14.5 morphometrics analysis**

| **Landmark Number** | **Landmark Definition** |
| --- | --- |
| 1 | Most rostral midline point on the developing rostrum |
| 2 | The ventral most midline point along the developing lip |
| 3 (15) | Dorso-caudal corner of the whisker field, taken on the skin right next to the plateau of the whisker field, rather than on the field itself |
| 4 (16) | Ventro-rostral tip of the plateau on the ventro-rostral member of the supra-orbital vibrissae pair that is found dorsal to the eye |
| 5 (17) | Rostral apex of the forming Medial Canthus of the eye |
| 6 (18) | Caudal apex of the forming Lateral Canthus of the eye |
| 7 (19) | Center of the infraorbital vibrissa found ventral to the eye |
| 8 (20) | Point at the rostral base of the dorso-caudal portion of the developing pina of the ear |
| 9 (21) | Point at the rostral base of the ventro-rostral portion of the developing pina of the ear |
| 10 (22) | Point at the edge of the whisker margin between the second and third whisker rows, counting from the top. This point is frequently next to the second large mystacial vibrissa. |
| 11 (23) | Ventro-caudal corner of the whisker field, taken on the skin right next to the plateau of the whisker field, rather than on the field itself |
| 12 (24) | Medial point on edge of nasal aperture at the point of inflection between the lower vertical portion and the upper diagonal portion of the nasal aperture |
| 13 (25) | Point at dorso-lateral most extent of nasal aperture |
| 14 (26) | Caudo-lateral most point on the upper lip, where it meets the lower lip |
