# Supplementary material for: Aberrant cell segregation in the craniofacial primordium and the emergence of facial dysmorphology in craniofrontonasal syndrome
Source: PLoS Genet. 2020 Feb 24;16(2):e1008300. doi: 10.1371/journal.pgen.1008300 (PMC7058351; doi:10.1371/journal.pgen.1008300)
Supplement: Attachment — Submitted filename: Response to reviewers.docx [file pgen.1008300.s016.docx]

We are grateful for the reviewers’ enthusiasm for our study, which was found to be a “scientifically rigorous and novel study that makes a significant contribution to the mechanistic understanding of craniofacial dysmorphology in CFNS”. We also appreciate their attention to detail and quantitative perspective, as well as their thoughtful consideration of larger questions beyond the existing study that we present here. In response to these reviews we have performed extensive additional experiments to enable quantitative analysis of cell sorting and to characterize the expression of EPHRIN-B1 and EPHB receptors in our different genetic models, including newly collected *Ephb1-3* compound mutant embryos lacking *Efnb1* mutant alleles. We have also added to and revised the manuscript to more explicitly communicate our conclusions and outstanding questions. We hope that the reviewers will agree that these changes have led to a much-improved, more quantitative and more precise manuscript. Responses to specific suggestions are listed below, and changes to the text that are pertinent to these specific suggestions are highlighted in red in the revised manuscript.

**Reviewer 1**

1. **(Tables 1-2 and 4-7) Define Df, SS, MS, etc.**

We have defined these abbreviations in the legend of Table 1 as the abbreviations are the same in each of these tables

2. **(Lines 195-197) Sentence needs to be more clearly stated.**

We have clarified this sentence by separating it into two sentences that more explicitly connect it with the supporting results. We have also changed the order to flow more logically. The new sentences can be found at lines 200-202.

3. **(Line 224) It needs to be stated that ephrinB1 expression was detected by IF.**

We have made this suggested change (line 227-228).

4. (**Lines 219-231) The ‘n’ for these experiments should be given here and throughout the main text.**

As suggested we have added n for all experiments integrated into the text when results are described. In addition, we provide Table S1, which includes genetic crosses and n for all experiments described in the manuscript.

5. **Quantitative analysis of the ephrinB1-negative (and/or positive) patches induced by Sox10-Cre mediated deletion at E10.5 vs. E11.5 vs. E13.5 would more strongly support the conclusion that an increase in the severity of cell-segregation is linked to an increase in the severity of the phenotype.**

We have now quantitatively analyzed cell segregation in every possible instance by two methods adapted from our previous manuscript (O’Neill et al., JCB, 2016). First, we measured the area of XGFP-positive patches as a measure of the extent of cell sorting, wherein small patches typically represent limited cell sorting, whereas larger patches represent the greater occurrence of cell sorting. Second, we counted the number of cells in patches to determine the distribution of patches with a range of different cell numbers in each of the mutant genotypes. We think that together these measures best quantify extent of cell segregation, and we have therefore applied this analysis to all segregation experiments in the manuscript. For the most part, this quantitative analysis supported our previous qualitative judgements. The exception is the extent of sorting in *Efnb1^+XGFP/lox^; Shox2^IresCre/+^* embryos, for which the difference in patch size from *Efnb1^+XGFP/lox^* control embryos was not statistically significant for the non-parametric statistical testing we performed. We have therefore modified the text to reflect this change (lines 275-282), which does not otherwise affect our conclusions.

6. **(Lines 263-265) Again, quantitative analysis of the ephrinB1-negative (and/or positive) patches would strengthen the correlation between cell segregation and the severity of the phenotype.**

We have performed this quantification, see response to 5.

7. **The ephrinB1 hemizygous males show facial dysmorphology that is similar (albeit, less severe) to the heterozygous females. Cell segregation via X-inactivation cannot account for the males’ phenotype. Therefore, the model that functional mosaicism in cranial mesenchyme induces dysmorphology does not seem to explain the craniofacial phenotype in males. Is it expected that the males’ phenotype results from loss of ephrinB1 function rather than functional mosaicism? This should be discussed in more detail.**

The reviewer is correct that the cell segregation as a consequence of X-inactivation mosaicism cannot explain *Efnb1* hemizygous dysmorphology. We think that normal function of ephrin-B1 includes effects on cell position in the craniofacial mesenchyme, but the XGFP transgene is not useful in addressing this question and it is not possible to characterize these effects using our existing genetic tools. Ongoing experiments in our lab seek to address whether and how ephrin-B1 controls normal cell positioning, which may result in the stereotyped effects on face shape based on normal ephrin-B1 expression boundaries. We have added text to the discussion (Lines 444-459) to share our hypotheses on this unanswered question.

8. **Figure S5- Text says there is no Sox1-Cre activation in the palatal shelves, but GFP+ cells are seen.**

We apologize for this imprecision. We do consistently observe a few Sox1-Cre lineage cells in the mesenchyme of both the secondary palate and FNP. We have revised the text (lines 297-300) to say that we observe “recombination in relatively few sparse mesenchymal cells in craniofacial structures such as the palatal shelves and FNP”, and now provide two examples of the recombination we observe in Fig. S5.

This low recombination is consistent with the absence of cell segregation in these embryos, though we do not know the minimum number of recombining cells that would allow cell segregation.

**9. (Lines 347-353) Cell segregation differences observed in the quad EphB receptor/ephrinB1+/delta allelic series should be measured in order to strengthen the conclusion that the abnormal cell sorting phenotype is partially rescued by KO of Ephb2 and Ephb3.**

Similar to the approach we apply in response to #5 and #6, we have now performed quantification of cell sorting in compound mutants by two methods. First, we measured the area of ephrin-B1 negative patches as a measure of the extent of cell sorting, wherein small patches typically represent limited cell sorting, whereas larger patches represent the greater occurrence of cell sorting. Second, we counted the number of cells in patches to determine the distribution of patches with a range of different cell numbers in each of the mutant genotypes. These data are now presented as additional panels in Figure 7, Figure S6 and Figure S7 and discussed in the text and methods.

**10. (Lines 388-389) Hemizygous males may exhibit ectopic cell segregation or loss of normal cell segregation not visible/appreciated by the techniques used here (X-GFP and ephrinB1 expression). For example, loss of ephrinB1 signaling may change Eph-ephrin signaling preferences that result in abnormal boundary establishment or weakening of normal boundaries.**

This is an excellent point. Indeed, we think that loss of ephrin-B1 does result in loss of normal cell distribution that is not revealed here and that may contribute to the stereotyped change in morphology that we observe (see also response to 7). We are currently developing strategies to attempt to characterize cell distribution in the absence of ephrin-B1, but in this manuscript have focused on CFNS-related heterozygous severity. However, new additional discussion text (lines 452-460) introduces and discusses this hypothesis.

**11. Figure 6 shows that the compound EphB receptor KOs exhibit hypertelorism, frontonasal dysplasia, and cleft pallet (phenotypes also induced by ephrinB1 mosaicism). Figure 7 shows that the quad EphB receptor/ephrinB1+/delta allelic series has varying severity in cell segregation depending on the EphB receptors deleted. To more completely understand the significance of the cell segregation phenotype, ephrinB1 IF should be carried out in the compound EphB receptor KOs.**

As suggested we have now performed EPHRIN-B1 IF in *Ephb1^-/-^*; *Ephb2^-/-^*; *Ephb3^-/-^* and other compound mutant embryos (Figure S7, lines 335-339). EPHRIN-B1 expression appears generally unperturbed, though changes in secondary palate shape in *Ephb* compound mutant embryos truncate the normal region of EPHRIN-B1 expression.

This result does not support changes in EPHRIN-B1 cell position in *Ephb* compound mutants, but we do not think this result rules out this possibility as regulative changes in EPHRIN-B1 expression may occur as cells change their position.

**Reviewer 2**

1. **The authors suggested that the mosaic loss of Efnb1 in Efnb1 heterozygous mutant mice leads to additional phenotypes not seen in Efnb1 hemizygous male mutant mice, caused by cell segregation. What are the cellular and molecular changes caused by mosaic loss of Efnb1 that lead to the cell segregation? Are there any specifically affected cell adhesion molecule or cell migration changes in Efnb1 heterozygous mutant mice?**

This is an excellent question, and one that we are very interested in answering. The cellular mechanisms that generally drive Eph/ephrin mediated cell segregation have been an area of extensive investigation for several research labs for many years. Our previous publication suggests that cell segregation involves changes in actomyosin distribution (O’Neill et al., JCB, 2016). We have pursued this initial observation, leading to a separate manuscript that we are currently preparing for publication. In that manuscript, we utilized biophysical approaches to discover that changes in cortical actomyosin contractility impact the strength of cell-cell contacts between Eph- and ephrin- expressing cells. This extensive cellular mechanistic study and the related functional genetic tests of this hypothesis go beyond the scope of the current manuscript, however.

**2. What happens to the non-Efnb1-expressing and Efnb1-expressing cells after cell segregation during development? Do these cells have different rates of proliferation and/r apoptosois after cell segregation? How do those cellular and molecular changes give rise to the observed changes in tissue shape such as bending, folding and bifurcation in the secondary palate and FNP that correlated with ectopic Efnb1 expression boundaries?**

We have now performed cleaved caspase 3 IF on control and *Efnb1* hemizygous and heterozygous mutants and find very few cells undergoing apoptosis in EPHRIN-B1 positive or negative domains in any genotype. Based on the low number of apoptotic cells, we do not attempt to quantify this result.

We have previously quantified cell proliferation rate in *Efnb1* mutant and control cells and found that indeed, EPHRIN-B1 signaling does impact cell proliferation rate, which may contribute to local and global phenotypes that we observe. We now discuss these data in our revised discussion (Iines 437-452).

**3. RNAseq analysis should be performed to compare the different gene expression profiles of wildtype, Efnb1 heterozygous female mutants, homozygous female mutants and Efnb1 hemizygous male mutants to identify the specific downstream changes caused by cell segregation compared to the complete loss of Efnb1.**

This is an excellent suggestion and one that we will carry out in future experiments. We have previously performed microarray analysis of palatal mesenchyme cells following induction with EPHRIN-B1 (Bush and Soriano, G&D, 2010). This experiment revealed an activation of a few Ras/MAPK immediate early targets, but relatively few genes exhibiting changes in expression. There may well be additional transcriptional changes *in vivo*, and particularly in *Efnb1^+/-^* embryos. We think though that the functional validation required to investigate the significance of transcriptional changes will go beyond the scope of the current manuscript, and we will instead pursue this investigation in a future study.

**4. The authors have shown that compound knockout of EphB receptors dramatically reduce cell segregation in Efnb1+/- mutants. Where are those three EphB receptors expressed? Is there any tissue-specific distribution of each different EphB receptor? Could this also explain why different combinational knockouts of EphB receptors reduce cell segregation differently, apart from the interactions between different EphB receptors?**

We have now performed IF examination of EPHB2 and EPHB3 receptor expression and RNA scope to detect the *Ephb1* transcript, for which we do not have a working antibody for IF (Fig. S6). We find that each receptor is expressed in tissues relevant to the cell segregation we described, though with varying apparent relative levels of expression in different contexts. Low level of *Ephb1* expression in the distal secondary palate mesenchyme does seem to fit with its apparently less important role in cell segregation in this context, but it also appears low in the telencephalon, where its loss does impact cell segregation. It is important to consider, however, that EPHRIN-B1 is expressed in the neuroepithelium from E8.5, and we do not determine the temporal competence of EPHRIN-B1 to drive cell segregation within the neuroepithelium in this study. We discuss these findings in (lines 331-335, and lines 486-487)

Minor comments:
**Fig. 1, Fig. S1, Fig. S2 need scale bars.**

We apologize for this oversight and have now added scale bars on these panels.
